# Overexpression of TOLLIP Protects against Acute Kidney Injury after Paraquat Intoxication through Inhibiting NLRP3 Inflammasome Activation Modulated by Toll-Like Receptor 2/4 Signaling

**DOI:** 10.1155/2021/5571272

**Published:** 2021-07-14

**Authors:** Qiang Zheng, Hang Zhao, Dong Jia, Xu Han, Zhenning Liu, Min Zhao

**Affiliations:** Department of Emergency Medicine, Shengjing Hospital of China Medical University, Shenyang, 110004 Liaoning Province, China

## Abstract

Paraquat (PQ) can cause multiorgan failure including acute kidney injury (AKI). Our prior study showed that Toll-interacting protein (TOLLIP) protected against PQ-induced acute lung injury. However, the role of TOLLIP in PQ-induced AKI remains undefined. This study was aimed at understanding the role and mechanism of TOLLIP in AKI. Six-eight-week-old male Wistar rats were intraperitoneally injected with 25 mg/kg PQ to induce AKI for 24 h *in vivo*. HK-2 cells were treated with 300 *μ*M PQ for 24 h to induce cellular injury *in vitro* or 300 *μ*M PQ and 5 *μ*M nuclear factor-*κ*B (NF-*κ*B) inhibitor BAY11-7082 for 24 h. Rats were infected with adenovirus carrying TOLLIP shRNA via tail vein injection and HK-2 cells with adenovirus carrying TOLLIP shRNA or TOLLIP 48 h before PQ exposure. Results showed that TOLLIP and Toll-like receptor 2/4 (TLR2/4) expressions were boosted in the kidney after PQ intoxication. The toxic effect of PQ on the kidney and HK-2 cells was exacerbated by TOLLIP knockdown, as evidenced by aggravated glomerulus and tubule injury, inflammatory infiltration, and cell apoptosis in the kidney and increased loss of cell viability and apoptotic cells in HK-2 cells. TOLLIP knockdown also enhanced PQ-induced NLR family pyrin domain-containing 3 (NLRP3) inflammasome activation *in vivo* and *in vitro* and TLR2/4-NF-*κ*B signaling *in vitro*, reflected by increased contents of proinflammatory cytokines and expressions of NLRP3 inflammasome-related proteins in the kidney and HK-2 cells and expressions of TLR2, TLR4, and nuclear NF-*κ*B p65 in HK-2 cells. However, TOLLIP overexpression inhibited PQ-induced loss of cell viability, cell apoptosis, NLRP3 inflammasome activation, and TLR2/4-NF-*κ*B signaling *in vitro*. Additionally, BAY11-7082 abolished TOLLIP knockdown-induced NLRP3 inflammasome activation *in vitro*, indicating that TOLLIP protected against NLRP3 inflammasome activation in PQ-induced AKI through inhibiting TLR2/4-NF-*κ*B signaling. This study highlights the importance of TOLLIP in AKI after PQ intoxication.

## 1. Introduction

Paraquat (also known as methyl viologen, PQ) has been around as a widely used herbicide in agricultural production due to its ability in high-efficiency nonselective killing of leaf weeds and plants since the early 1960s [1]. However, growing evidence has pointed out its harmfulness to the mammals including human, rodents, and rabbits [2, 3]. PQ intoxication is a severe threat to human health in developing countries, especially in Asia. No specific antidote has been utilized to treat PQ intoxication so far. Exposure to PQ causes severe damage in the various organ systems including the lungs, kidney, and liver [4]. The lung is the main target organ in PQ intoxication, and the respiratory failure caused by acute lung injury is the most usual cause of death in PQ intoxication patients [5]. Additionally, PQ is mainly enriched in the kidney at 3 h after exposure, which resulted in acute kidney injury (AKI) [6]. The mechanism underlying AKI after PQ intoxication, however, has not been fully understood, leading to a limitation of appropriate treatment strategy.

Toll-like receptors (TLRs), the first pattern recognition receptors, play a pivotal role in triggering innate immune responses. Activated TLRs recruit myeloid differentiation factor 88 (MyD88) via its Toll-interleukin-1 receptor (IL-1R) domain [7], and then, MyD88 binds to IL-1R-associated kinase (IRAK) via N-terminal death domain [8]. Subsequently, IRAK undergoes phosphorylation and activation, resulting in the activation of various downstream signaling pathways including nuclear factor-*κ*B (NF-*κ*B) and extracellular signal-regulated kinase pathways [9]. Accumulating evidence has shown that TLRs play a key role in the pathogenesis of multiple diseases including cerebral hemorrhage [10], atherosclerosis [11], nonalcoholic fatty liver disease [12], and pulmonary fibrosis [13]. TLR-mediated signaling pathway is also involved in AKI, and inhibition of this signaling has been shown to attenuate AKI [14].

Toll-interacting protein (TOLLIP), a multifunctional protein, consists of a Tom1-binding domain in the N-terminus, a conserved 2 domain in the central region, and a coupling of ubiquitin conjugation to endoplasmic reticulum degradation domain in the C-terminus in mammals [15]. TOLLIP has been identified as a negative modulator of TLR signaling. TOLLIP interacts with TLR2 and TLR4 by binding to TLR2 and the TLR4-myeloid differentiation factor 2 complex via its C-terminus, thus suppressing TLR-mediated cellular responses by dampening the phosphorylation and activity of IRAK [16]. In addition, TOLLIP also serves as a regulator of protein sorting via its interaction with Tom1, ubiquitin, and clathrin [17]. Our prior research showed that TOLLIP was aberrantly expressed in the lung and TOLLIP overexpression exerted a protective effect on lung injury after PQ intoxication [18]. TOLLIP is also expressed in the kidney; however, whether TOLLIP is involved in AKI after PQ intoxication remains unknown.

In the present study, the expression of TOLLIP, TLR2, and TLR4 was analyzed in rats after PQ intoxication. To better understand the role of TOLLIP in AKI after PQ intoxication, the expression of TOLLIP in rats and HK-2 cells was knocked down or overexpressed and the effect of TOLLIP knockdown or overexpression on the renal or cellular injury and NLR family pyrin domain-containing 3 (NLRP3) inflammasome *in vivo* and *in vitro* was subsequently investigated. In addition, it was also verified whether the TLR2/4-NF-*κ*B pathway was implicated in the protective mechanism of TOLLIP in PQ-induced AKI. This study provides a potential target for PQ-induced AKI treatment.

## 2. Materials and Methods

### 2.1. Animals

Male Wistar rats aged 6-8 weeks were purchased from Liaoning Changsheng Biotechnology Co., Ltd. (China). All rats were housed at 22 ± 1°C under 12 h/12 h light/dark cycles and were given free access to food and water.

### 2.2. Animal Experimental Protocol

In some experiments, animals were divided randomly into 2 groups (Sham and PQ groups). Rats in the PQ group were intraperitoneally injected with 25 mg/kg PQ (Aladdin, China), while rats in the Sham group were intraperitoneally injected with an equal volume of normal saline. Rats in the PQ group were euthanatized at 3, 6, 12, and 24 h after PQ injection. Rats in the Sham group were euthanatized at 24 h after saline injection. The kidney tissues were collected for further analysis.

In some experiments, animals were divided randomly into 4 groups (Sham, PQ, PQ+adenovirus- (AV-) negative control short hairpin RNA (shNC), and PQ+AV-TOLLIP short hairpin RNA (shTOLLIP) groups). Forty-eight h before PQ injection, rats in the PQ+AV-shNC and PQ+AV-shTOLLIP groups were infected with the AV carrying shTOLLIP or shNC (10^9^ PFU, tail vein injection). Rats in the PQ, PQ+AV-shNC, and PQ+AV-shTOLLIP groups were intraperitoneally injected with 25 mg/kg PQ, while rats in the Sham group were intraperitoneally injected with an equal volume of normal saline. All rats were euthanatized at 24 h after injection. The kidney tissues and serum were collected for further analysis.

All animal experiments were performed following the *Guide for the Care and Use of Laboratory Animals* [19]. All procedures were reviewed and approved by the ethics committee of animal use at Shengjing Hospital of China Medical University (Accession number, 2020PS702K).

### 2.3. RNA Isolation and Real-Time Quantitative Reverse Transcription PCR (Real-Time qRT-PCR)

Total RNA was isolated from the kidney tissues using TRIpure (BioTeke, China) according to the manufacturer's instruction and reversely transcribed to generate cDNA. Real-time qPCR was performed using SYBR Green (Solarbio, China) and 2× Taq PCR MasterMix (Solarbio). The following primers were used: TOLLIP, 5′-CAGCCTGTGGTTCTGATG-3′ (forward) and 5′-TCTTTGTTCCCTCTTTGG-3′ (reverse); TLR2, 5′-TATTCTGAGTTCCGTGAG-3′ (forward) and 5′-TTACCGTTTCTACTTTACC-3′ (reverse); TLR4, 5′-AATCTGGTGGCTGTGG-3′ (forward) and 5′-TGGGCTTGAATGGAGT-3′ (reverse); *β*-actin, 5′-GGAGATTACTGCCCTGGCTCCTAGC-3′ (forward) and 5′-GGCCGGACTCATCGTACTCCTGCTT-3′ (reverse). *β*-Actin was utilized as a housekeeping reference gene.

### 2.4. Histopathological Analysis

The kidney tissue was embedded in paraffin. The paraffin-embedded kidney sections (5 *μ*m) were deparaffinated in xylene and stained with hematoxylin (Solarbio) and eosin (Sangon) (H&E). Histopathological changes of the kidney tissues were observed under a microscope (OLYMPUS, Japan).

### 2.5. Measurement of Blood Urea Nitrogen (BUN) and Serum Creatinine Level

The BUN and serum creatinine levels in rats were measured by their respective commercial kits (Nanjing Jiancheng, China) in accordance with the manufacturer's protocol.

### 2.6. Cell Culture and Experimental Protocol

HK-2 cells, a proximal tubular cell line derived from normal kidney, retain functional characteristics of proximal tubular epithelium. HK-2 cells were purchased from Procell Life Science & Technology Co., Ltd. (China), and cultured in Minimum Essential Medium (MEM, Procell) supplemented with 10% fetal bovine serum (TIANHANG, China) in a humidified 37°C incubator with 5% CO_2_. HK-2 cells were infected with the AV carrying shTOLLIP or TOLLIP and treated with 300 *μ*M PQ for 24 h at 48 h postinfection at 37°C and 5% CO_2_. HK-2 cells were infected with the AV carrying shTOLLIP and treated with 300 *μ*M PQ and 5 *μ*M NF-*κ*B inhibitor BAY11-7082 (Beyotime, China) for 24 h at 48 h postinfection at 37°C and 5% CO_2_.

### 2.7. Immunofluorescence Staining

The kidney tissue sections (5 *μ*m) were deparaffinized, rehydrated, and washed with PBS. Cells were fixed in 4% paraformaldehyde, incubated with 0.1% Triton X-100 (Beyotime), and washed with PBS. Tissue sections and cells were blocked in goat serum for 15 min. For TOLLIP-TLR2 and TOLLIP-TLR4 staining, tissue sections were subjected to incubation with anti-TOLLIP (ABclonal, China) and anti-TLR2 (NOVUS, USA)/anti-TLR4 (Santa Cruz, USA) antibodies (1 : 50 dilution) at 4°C overnight, followed by incubation with FITC-conjugated goat anti-rabbit IgG or Cy3-conjugated goat anti-mouse IgG (Beyotime; 1 : 200 dilution) at room temperature for 90 min. For cleaved caspase-3, NLRP3, and p65 staining, tissue sections or cells were subjected to incubation with anti-cleaved caspase-3 (Affinity, China), anti-NLRP3 (ABclonal), and anti-p65 (Affinity) antibodies (1 : 100 dilution) at 4°C overnight, followed by incubation with Cy3-conjugated goat anti-rabbit IgG (1 : 200 dilution) at room temperature for 60 min. Next, tissue sections or cells were subjected to dihydrochloride (DAPI) (Aladdin) nuclear staining. Images were captured using a fluorescence microscope (OLYMPUS).

### 2.8. Western Immunoblot Analysis

Total protein was isolated from the kidney tissues or cells using cell lysis buffer for Western and IP containing 1 mM phenylmethylsulfonyl fluoride (Beyotime). Nuclear protein was isolated from cells using a Nuclear and Cytoplasmic Protein Extraction Kit (Beyotime) following the manufacturer's protocol. Approximately 20 to 40 *μ*g of protein sample was resolved by sodium dodecyl sulfate polyacrylamide gel electrophoresis and transferred to a polyvinylidene fluoride membrane. Blots were blocked at room temperature for 1 h. Next, the blots were incubated overnight at 4°C with primary antibodies including anti-TOLLIP antibody (ABclonal; 1 : 1000 dilution), anti-NLRP3 antibody (ABclonal; 1 : 1000 dilution), anti-apoptosis-associated speck-like protein containing a caspase recruitment domain (ASC) antibody (ABclonal; 1 : 1000 dilution), anti-pro/mature caspase-1 antibody (ABclonal; 1 : 1000 dilution), anti-cleaved caspase-3 antibody (Affinity; 1 : 1000 dilution), anti-cleaved poly (adenosine diphosphate ribose) polymerase (PARP) antibody (CST, USA; 1 : 1000 dilution), anti-TLR2 antibody (NOVUS; 1 : 2000 dilution), anti-TLR4 antibody (Santa Cruz; 1 : 300 dilution), anti-p65 antibody (Affinity; 1 : 1000 dilution), anti-*β*-actin antibody (Santa Cruz; 1 : 1000 dilution), and anti-Histone H3 antibody (ABGENT, USA; 1 : 2000 dilution). Then, the blots were incubated with HRP-conjugated goat anti-rabbit IgG or HRP-conjugated goat anti-mouse IgG (Beyotime; 1 : 5000 dilution) for 45 min at 37°C. *β*-Actin and Histone H3 were used as an internal reference. The intensity of blots was analyzed using Gel-Pro Analyzer software.

### 2.9. Detection of Cytokine Level

The contents of tumor necrosis factor *α* (TNF-*α*), interleukin-1*β* (IL-1*β*), interleukin-6 (IL-6), and interleukin-18 (IL-18) in the kidney tissues or cells were quantified using their respective enzyme-linked immunosorbent assay kits (MultiSciences, China; Fine Test, China) in accordance with the manufacturer's protocol.

### 2.10. Cell Counting Kit-8 (CCK-8) Assay

HK-2 cells (5 × 10^3^ per well) were seeded in a 96-well culture plate. After treatment, cell viability was measured using CCK-8 (KeyGEN, China). Briefly, 10 *μ*L CCK-8 solution was added to each well and incubated for 2 h at 37°C. The absorbance was measured at 450 nm using a microplate reader (BIOTEK, USA).

### 2.11. Detection of Cell Apoptosis

Apoptotic cells were stained using an Annexin V-Fluorescein (FITC)/propidium iodide (PI) apoptosis kit (KeyGEN) according to the manufacturer's instruction and detected by a NovoCyte flow cytometer (Aceabio, USA). Apoptotic cells were the sum of early apoptotic cells (Annexin V-FITC^+^/PI^−^) and late apoptotic cells (Annexin V-FITC^+^/PI^+^).

### 2.12. Statistical Analysis

Data are expressed as mean ± SD and compared by one-way analysis of variance (ANOVA). When *p* < 0.05, differences were considered statistically significant. Statistical analysis was conducted by GraphPad Prism 8.0.

## 3. Results

### 3.1. TOLLIP Is Upregulated in the Rat Kidney in PQ-Induced AKI

**Figure 1:**
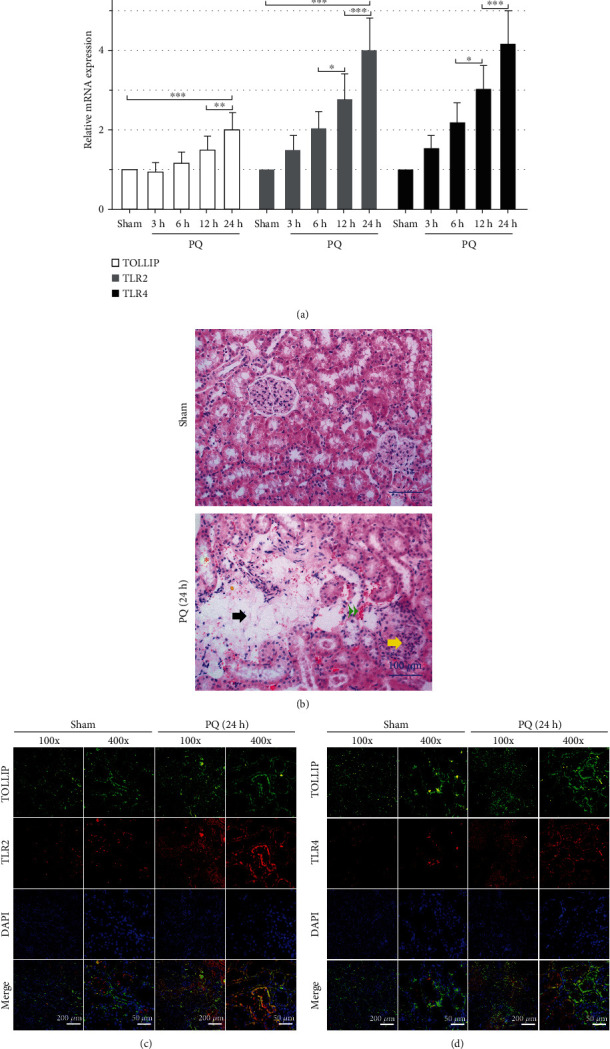
Effect of PQ on the expression of TOLLIP, TLR2, and TLR4 in the rat kidney tissues. Wistar rats received 25 mg/kg PQ via intraperitoneal injection. (a) The mRNA expression of TOLLIP, TLR2, and TLR4 in the kidney tissues at 3, 6, 12, and 24 h post-PQ treatment. (b) Representative images of H&E staining of the kidney tissues at 24 h post-PQ treatment (red asterisk, degenerative renal tubules; black arrow, necrosis; yellow arrow, degenerative glomerulus; and green double arrowhead, hemorrhage). (c, d) Representative images of TLR2-TOLLIP and TLR4-TOLLIP staining of the kidney tissues at 24 h post-PQ treatment. ^∗^*p* < 0.05, ^∗∗^*p* < 0.01, and ^∗∗∗^*p* < 0.001 (*n* = 8).

In the rats of the PQ group, the mRNA expression levels of TOLLIP, TLR2, and TLR4 in the kidney increased gradually during PQ exposure (Figure 1(a)). In comparison with the rats of the Sham group, the mRNA expression levels of TOLLIP, TLR2, and TLR4 in the kidney were significantly increased by PQ exposure (Figure 1(a)). As shown in Figure 1(b), the rats of the Sham group showed the normal structure of the glomerulus and renal tubule, while the rats of the PQ group showed histopathological alterations in the kidney including necrosis, hemorrhage, and degenerative glomerulus and renal tubules. It has been reported that TOLLIP interacted with TLR2 and TLR4 by binding to TLR2 and TLR4 via its C-terminus [20]. Hence, TOLLIP-TLR2 and TOLLIP-TLR4 staining was performed to verify whether the interaction between TOLLIP and TLR2/TLR4 existed in the pathogenesis of PQ-induced AKI. As shown in Figures 1(c) and 1(d), less stained area for TOLLIP and TLR2/4 in the Sham group was overlapped compared with that in the PQ group, indicating that PQ might result in more interaction between TOLLIP and TLR2/4 in the kidney tissues.

### 3.2. Knockdown of TOLLIP Exacerbates PQ-Induced AKI in Rats

**Figure 2:**
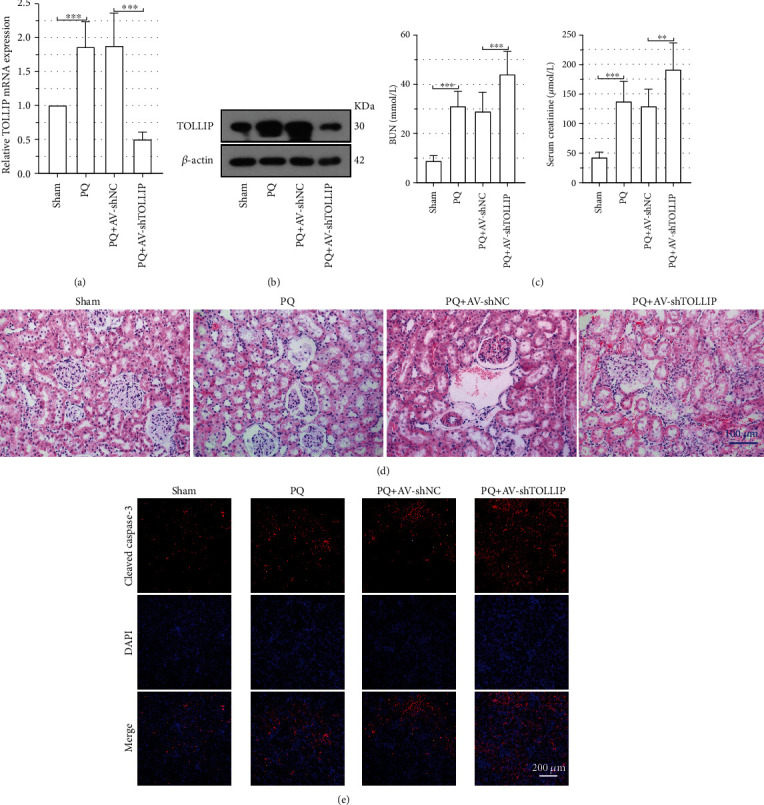
Effect of TOLLIP knockdown on the PQ-induced AKI in rats. Wistar rats were infected with the AV carrying shTOLLIP via tail vein injection and then treated with 25 mg/kg PQ via intraperitoneal injection 48 h post-AV infection. (a, b) The mRNA and protein expression of TOLLIP in the kidney tissues at 24 h post-PQ treatment. (c) The level of BUN and creatinine in the serum at 24 h post-PQ treatment. (d) Representative images of H&E staining of the kidney tissues at 24 h post-PQ treatment. (e) Representative images of cleaved caspase-3 immunofluorescence staining of the kidney tissues at 24 h post-PQ treatment. ^∗∗^*p* < 0.01 and ^∗∗∗^*p* < 0.001 (*n* = 8).

In order to determine whether TOLLIP was involved in PQ-induced AKI, rats were injected with the AV carrying shTOLLIP via the tail vein prior to PQ exposure. The mRNA and protein expression levels of TOLLIP in the kidney from the rats of the PQ+AV-shTOLLIP group were significantly decreased (Figures 2(a) and 2(b)). Knockdown of TOLLIP resulted in significant increases in BUN and serum creatinine levels compared to the PQ+AV-shNC group (Figure 2(c); PQ+AV-shTOLLIP vs. PQ+AV-shNC: BUN, 44.27 ± 9.45 mmol/L vs. 29.03 ± 7.76 mmol/L; serum creatinine, 190.87 ± 46.07 *μ*mol/L vs. 128.76 ± 29.32 *μ*mol/L). Furthermore, more severe glomerulus and tubule injury as well as inflammatory cell infiltration was displayed in the PQ+AV-shTOLLIP group (Figure 2(d)). Staining for cleaved caspase-3 (Figure 2(e)) showed that PQ exposure increased cleaved caspase-3 expression in the rat kidney but knockdown of TOLLIP further increased cleaved caspase-3 expression in the kidney. These data suggested that TOLLIP played a protective role in PQ-induced AKI.

### 3.3. Knockdown of TOLLIP Promoted the PQ-Induced Activation of NLRP3 Inflammasome in the Rat Kidney

**Figure 3:**
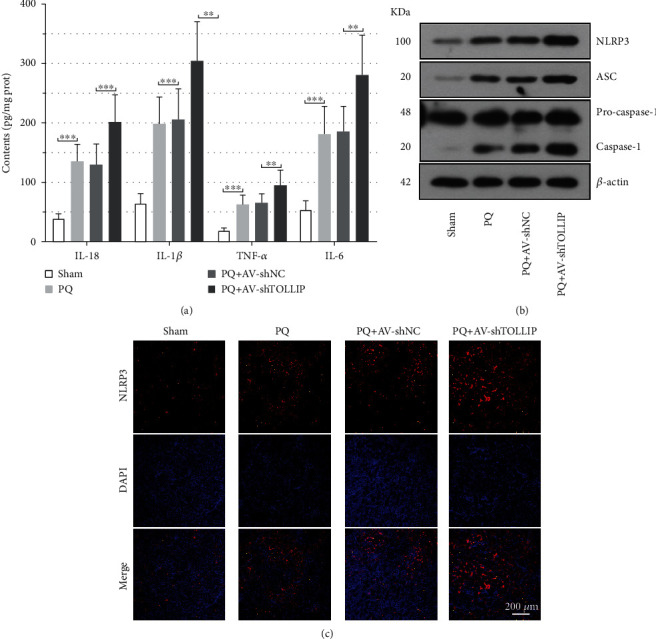
Effect of TOLLIP knockdown on the PQ-induced production of NLRP3 inflammasome in the rat kidney tissues. Wistar rats were infected with the AV carrying shTOLLIP via tail vein injection and then treated with 25 mg/kg PQ via intraperitoneal injection 48 h post-AV infection. (a) The level of IL-18, IL-1*β*, TNF-*α*, and IL-6 in the kidney tissues at 24 h post-PQ treatment. (b) The protein expression of NLRP3, ASC, pro-caspase-1, and caspase-1 in the kidney tissues at 24 h post-PQ treatment. (c) Representative images of NLRP3 immunofluorescence staining of the kidney tissues at 24 h post-PQ treatment. ^∗∗^*p* < 0.01 and ^∗∗∗^*p* < 0.001 (*n* = 8).

Subsequently, the effect of TOLLIP knockdown on the activation of NLRP3 inflammasome in the rat kidney was investigated. As shown in Figure 3(a), PQ exposure increased the level of IL-18, IL-1*β*, TNF-*α*, and IL-6, while knockdown of TOLLIP further increased their levels (PQ+AV-shTOLLIP vs. PQ+AV-shNC: IL-18, 202.21 ± 45.13 pg/mg prot vs. 129.74 ± 34.29 pg/mg prot; IL-1*β*, 305.35 ± 65.66 pg/mg prot vs. 205.65 ± 52.24 pg/mg prot; TNF-*α*, 95.77 ± 24.46 pg/mg prot vs. 65.95 ± 14.47 pg/mg prot; IL-6, 281.56 ± 66.41 pg/mg prot vs. 186.85 ± 40.39 pg/mg prot). Western immunoblot analysis for NLRP3 inflammasome-related proteins (Figure 3(b)) also exhibited further increased expression levels of NLRP3, ASC, and caspase-1 in the kidney from the rats of the PQ+AV-shTOLLIP group. Consistent with Western immunoblot analysis, staining for NLRP3 (Figure 3(c)) showed that knockdown of TOLLIP further increased the NLRP3 expression induced by PQ exposure in the kidney. These data indicated that TOLLIP inhibited the PQ-induced activation of NLRP3 inflammasome in the rat kidney.

### 3.4. TOLLIP Is Involved in the PQ-Induced Loss of HK-2 Cell Viability and Apoptosis of HK-2 Cells

**Figure 4:**
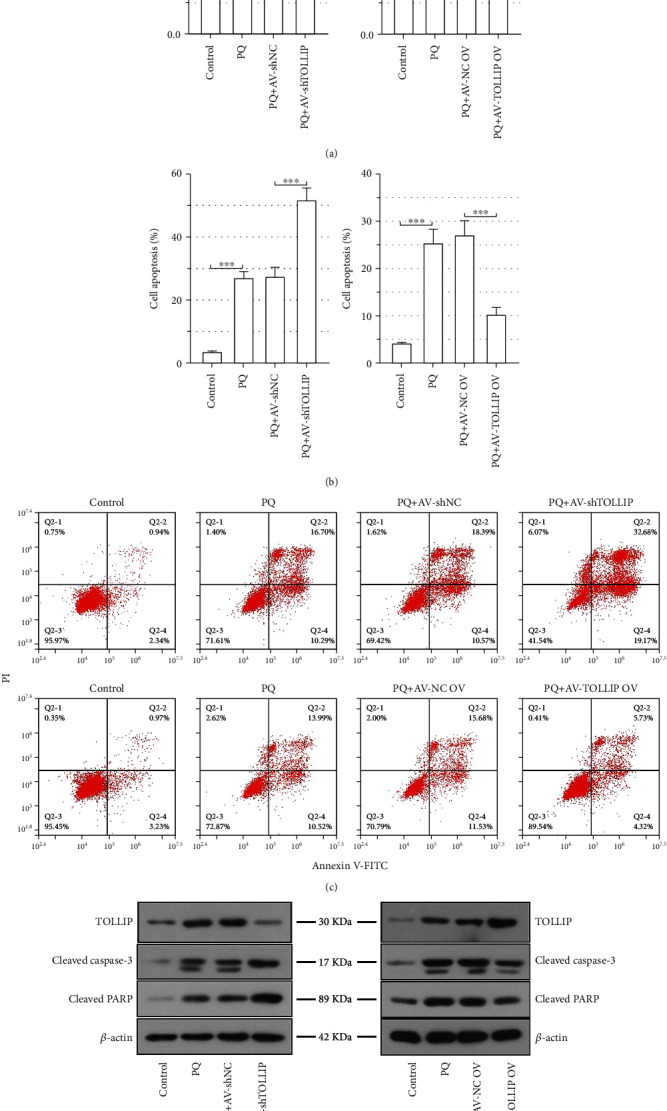
Effect of TOLLIP knockdown and overexpression on the PQ-induced loss of cell viability and apoptosis of HK-2 cells. HK-2 cells were infected with the AV carrying shTOLLIP or TOLLIP and then treated with 300 *μ*M PQ 48 h post-AV infection. (a) Cell viability was measured by CCK-8 assay at 24 h post-PQ treatment. (b, c) Cell apoptosis was determined by Annexin V-FITC-PI staining at 24 h post-PQ treatment. (d) The protein expression of TOLLIP, cleaved caspase-3, and cleaved PARP at 24 h post-PQ treatment. ^∗^*p* < 0.05, ^∗∗^*p* < 0.01, and ^∗∗∗^*p* < 0.001 (*n* = 3).

In order to investigate the role of TOLLIP in PQ-induced AKI *in vitro*, HK-2 cells were infected with the AV carrying shTOLLIP or TOLLIP prior to PQ treatment. PQ significantly decreased the viability of HK-2 cells (Figure 4(a)). The loss of cell viability was further promoted by knockdown of TOLLIP but reversed by overexpression of TOLLIP (Figure 4(a)). PQ was also observed to significantly increase the percentage of apoptotic cells (Figures 4(b) and 4(c)). The percentage of apoptotic cells in the PQ-stimulated cells was significantly increased by knockdown of TOLLIP but reduced by overexpression of TOLLIP (Figures 4(b) and 4(c); PQ+AV-shTOLLIP vs. PQ+AV-shNC: 51.50% ± 3.89% vs. 27.36% ± 3.01%; PQ+AV-TOLLIP OV vs. PQ+AV-NC OV: 10.36% ± 1.45% vs. 27.13% ± 3.11%). Western immunoblot analysis (Figure 4(d)) exhibited that PQ treatment promoted the expression of TOLLIP, cleaved caspase-3, and cleaved PARP in HK-2 cells and the upregulated expression of cleaved caspase-3 and cleaved PARP induced by PQ was promoted by knockdown of TOLLIP but inhibited by overexpression of TOLLIP. These data demonstrated that TOLLIP protected against PQ-induced HK-2 cell injury.

### 3.5. TOLLIP Regulated the PQ-Induced Activation of NLRP3 Inflammasome in HK-2 Cells via TLR2/4-NF-*κ*B Pathway

**Figure 5:**
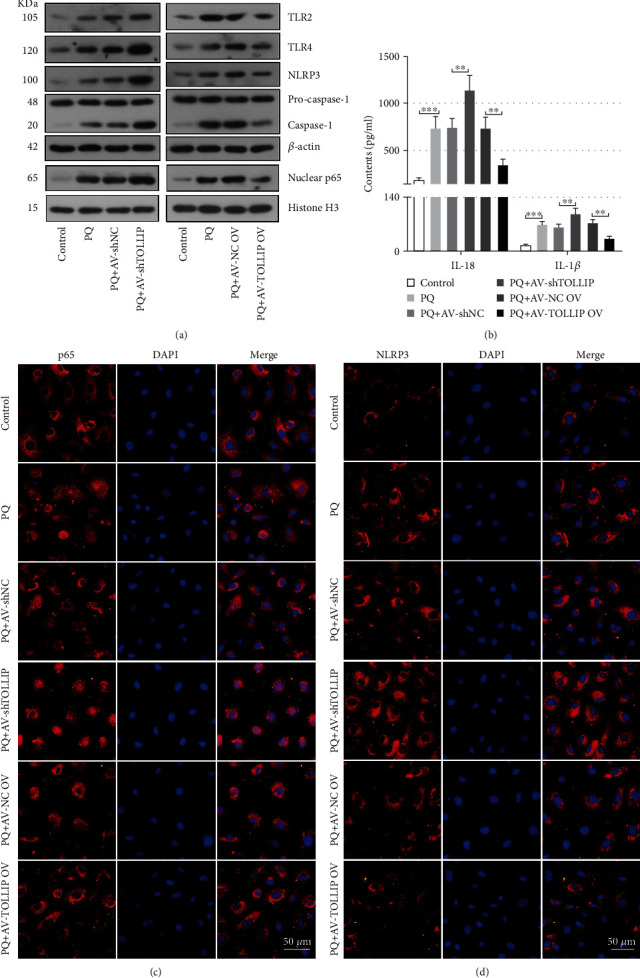
Effect of TOLLIP knockdown and overexpression on the PQ-induced activation of NLRP3 inflammasome in HK-2 cells. HK-2 cells were infected with the AV carrying shTOLLIP or TOLLIP and then treated with 300 *μ*M PQ 48 h post-AV infection. (a) The protein expression of TLR2, TLR4, NLRP3, pro-caspase-1, caspase-1, and nuclear p65 at 24 h post-PQ treatment. (b) The level of IL-18 and IL-1*β* at 24 h post-PQ treatment. (c, d) Representative images of p65 and NLRP3 immunofluorescence staining at 24 h post-PQ treatment. ^∗∗^*p* < 0.01 and ^∗∗∗^*p* < 0.001 (*n* = 3).

Next, the activation of NLRP3 inflammasome in HK-2 cells was analyzed. As shown in Figure 5(a), the expression of TLR2, TLR4, nuclear p65, NLRP3, and caspase-1 was increased by PQ exposure. Consistent with *in vivo* experiments, knockdown of TOLLIP further increased the expression of NLRP3 and caspase-1 in HK-2 cells (Figure 5(a)). However, overexpression of TOLLIP inhibited the PQ-induced expression of NLRP3 and caspase-1 in HK-2 cells (Figure 5(a)). Additionally, PQ-induced expression of TLR2, TLR4, and nuclear p65 was also promoted by knockdown of TOLLIP but inhibited by overexpression of TOLLIP (Figure 5(a)). As shown in Figure 5(b), knockdown of TOLLIP facilitated the PQ-induced increases in IL-18 and IL-1*β* content (PQ+AV-shTOLLIP vs. PQ+AV-shNC: IL-18, 1140.04 ± 160.03 pg/ml vs. 743.95 ± 96.86 pg/ml; IL-1*β*, 95.69 ± 15.02 pg/ml vs. 62.81 ± 7.67 pg/ml) in cell culture supernatant, but overexpression of TOLLIP suppressed their increases (PQ+AV-TOLLIP OV vs. PQ+AV-NC OV: IL-18, 344.85 ± 62.87 pg/ml vs. 730.17 ± 120.75 pg/ml; IL-1*β*, 32.95 ± 6.04 pg/ml vs. 72.78 ± 9.73 pg/ml). Staining for p65 (Figure 5(c)) revealed that PQ-induced p65 nuclear translocation in HK-2 cells was enhanced by knockdown of TOLLIP but blocked by overexpression of TOLLIP. Staining for NLRP3 (Figure 5(d)) showed an increased expression in the PQ+AV-shTOLLIP group and a reduced expression in the PQ+AV-TOLLIP OV group compared with the PQ+AV-shNC or PQ+AV-NC OV group.

**Figure 6:**
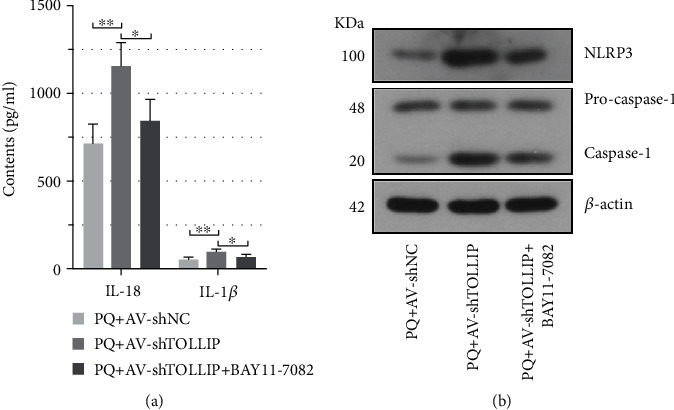
Role of the NF-*κ*B pathway in the TOLLIP-induced inhibition of NLRP3 inflammasome activation in PQ-treated HK-2 cells. HK-2 cells were infected with the AV carrying shTOLLIP and then treated with 300 *μ*M PQ and 5 *μ*M BAY11-7082 48 h post-AV infection. (a) The level of IL-18 and IL-1*β* at 24 h post-PQ and BAY11-7082 treatment. (b) The protein expression of NLRP3, pro-caspase-1, and caspase-1 at 24 h post-PQ and BAY11-7082 treatment. ^∗^*p* < 0.05 and ^∗∗^*p* < 0.01 (*n* = 3).

In order to explore whether the TLR2/4-NF-*κ*B pathway was implicated in TOLLIP-induced inhibition of NLRP3 inflammasome activation, HK-2 cells were infected with the AV carrying shTOLLIP and then treated with PQ and NF-*κ*B inhibitor BAY11-7082. BAY11-7082 reversed the increased level of IL-18 and IL-1*β* (PQ+AV-shTOLLIP+BAY11-7082 vs. PQ+AV-shTOLLIP: IL-18, 849.14 ± 117.61 pg/ml vs. 1160.03 ± 127.09 pg/ml; IL-1*β*, 76.64 ± 9.79 pg/ml vs. 102.04 ± 12.22 pg/ml) and expression of NLRP3 and caspase-1 caused by knockdown of TOLLIP (Figures 6(a) and 6(b)). Taken together, these data illustrated that TOLLIP inhibited PQ-induced activation of NLRP3 inflammasome through inhibiting the TLR2/4-NF-*κ*B pathway.

## 4. Discussion

PQ intoxication leads to a large amount of deaths, whose mortality rate is as high as 60%~80% [21]. The kidney is also a major target organ after PQ intoxication apart from the lung. Therefore, it is necessary to explore the mechanism underlying AKI after PQ intoxication.

It is widely accepted that TOLLIP is a negative regulator of inflammatory response. In our prior study, the expression of TOLLIP was downregulated in the lung in rats after PQ intoxication and TOLLIP overexpression mitigated PQ-induced acute lung injury [18]. In contrast to our prior study, the present study showed that the expression of TOLLIP was upregulated in the kidney after PQ intoxication. It has been reported that TOLLIP was increased in lung injury after cecal ligation and puncture [22] but decreased in hepatic ischemia-reperfusion injury [23]. Therefore, TOLLIP may exert different roles in the pathogenesis of different disorders. Subsequently, the effect of TOLLIP inhibition on AKI after PQ intoxication was analyzed in the present study. As a result, TOLLIP inhibition obviously aggravated kidney injury as evidenced by renal pathological changes using H&E staining. BUN and serum creatinine are commonly determined to evaluate renal function [24]. Increased levels of BUN and creatinine were found in the serum of PQ-poisoning patients [25]. The present study showed that PQ-induced increases in BUN and serum creatinine level were promoted by TOLLIP inhibition. These findings reflected loss of renal function by TOLLIP inhibition. Similar with our prior study, TOLLIP also showed a protective role in PQ-induced kidney injury.

PQ has been reported to trigger kidney cell apoptosis *in vivo* and *in vitro* [26, 27]. TOLLIP functions as a positive or negative regulator of cell apoptosis. TOLLIP has been demonstrated to inhibit bleomycin-induced bronchial epithelial cell apoptosis and IFN-*γ*- and TNF-*α*-induced intestinal epithelial cell apoptosis [28, 29]. However, TOLLIP could promote apoptosis of cardiomyocytes and hepatocytes [23, 30]. In the present study, TOLLIP inhibition increased the expression of cleaved caspase-3 in the kidney tissues and HK-2 cells and cleaved PARP expression in HK-2 cells as well as the percentage of apoptotic HK-2 cells, indicating increased PQ-induced kidney cell apoptosis by TOLLIP inhibition. In *in vitro* experiments, TOLLIP upregulation led to the opposite results. These finding indicated that TOLLIP protected kidney cells from PQ-induced apoptosis.

Aberrant activation of NLRP3 inflammasome is closely related to the development of various diseases, including inflammatory diseases [31], infections [32], Alzheimer's disease [33], and diabetes [34]. The NLRP3 inflammasome comprises the sensor NLRP3, the adaptor ASC, and the effector serine caspase-1 [35]. Multiple bacterial products or danger signals have been shown to induce activation of NLRP3 inflammasome [36]. Emerging evidence has elucidated that its activation was a contributor of renal inflammation, fibrosis, and ischemia-induced AKI [37, 38]. In recent years, excessive activation of NLRP3 inflammasome was found in the kidney after PQ intoxication [25]. Activation of NLRP3 inflammasome induces caspase-1 activation, which results in cleavage of pro-IL-1*β* and pro-IL-18 into their active forms IL-1*β* and IL-18 [39]. Consistent with a previous study, the present study showed that PQ increased the expression of NLRP3, ASC, and caspase-1 and secretion of IL-18, IL-1*β*, IL-6, and TNF-*α* in the kidney or HK-2 cells. Significantly, TOLLIP inhibition promoted the PQ-induced increases *in vivo* and *in vitro*. However, TOLLIP upregulation abrogated the PQ-induced increases *in vitro*. The findings first revealed the role of TOLLIP in activation of NLRP3 inflammasome in AKI. As reported, NLRP3 inflammasome induced cell apoptosis during kidney injury [40, 41]. Hence, TOLLIP-induced reduction in cell apoptosis during PQ intoxication might be attributed to blockage of NLRP3 inflammasome activation.

TOLLIP has been generally considered as a negative regulator of the TLR-mediated signaling pathway [42]. Activation of the TLR-mediated signaling pathway has been involved in the pathogenesis of multiple renal diseases, including fibrosis [43], ischemia/reperfusion injury [44], glomerulonephritis [45], and AKI [46]. Blockade of the TLR-mediated signaling pathway via MyD88 has been reported to mitigate lung injury after PQ intoxication [47]. In this present study, PQ enhanced the expression of TLR2 and TLR4 in the rat kidney. TOLLIP inhibition promoted PQ-induced expression of TLR2, TLR4, and nuclear p65 and p65 nuclear translocation *in vitro*, while TOLLIP upregulation eliminated the PQ-induced activation of TLR2/4 and its downstream NF-*κ*B signaling. The TLR2/4-mediated signaling pathway triggers the activation of NLRP3 inflammasome [48]. To confirm whether TLR2/4-mediated signaling was involved in TOLLIP-induced inhibition in NLRP3 inflammasome activation, NF-*κ*B inhibitor was utilized to treat HK-2 cells after TOLLIP knockdown. The NF-*κ*B inhibitor was found to reverse the activation of NLRP3 inflammasome induced by TOLLIP knockdown, indicating that the function of TOLLIP in NLRP3 inflammasome was at least partially mediated by TLR2/4-NF-*κ*B signaling. Besides, mitogen-activated protein kinase (MAPK) pathway was also a downstream signaling pathway of TLR signaling. Likewise, the MAPK signaling pathway induces the activation of NLRP3 inflammasome in kidney injury [49]. Therefore, it can be investigated in the future whether the MAPK pathway is implicated in PQ intoxication-caused activation of NLRP3 inflammasome and inflammatory cascades.

## 5. Conclusion

In summary, TOLLIP and TLR2/4 expressions were boosted in the kidney after PQ intoxication. TOLLIP inhibition exacerbated PQ-induced kidney and cellular injury and activation of NLRP3 inflammasome in rats and HK-2 cells and enhanced TLR2/4 and its downstream NF-*κ*B signaling pathway in HK-2 cells. However, TOLLIP upregulation had an opposite effect on PQ-induced cellular injury and activation of NLRP3 inflammasome as well as TLR2/4 and its downstream NF-*κ*B signaling pathway in HK-2 cells. In addition, the NF-*κ*B inhibitor restored the activation of NLRP3 inflammasome induced by TOLLIP inhibition upon PQ exposure in HK-2 cells. These data indicated that the protective role of TOLLIP against NLRP3 inflammasome activation in PQ-induced AKI was at least partially mediated by TLR2/4-NF-*κ*B signaling. This study highlights the importance of TOLLIP in AKI after PQ intoxication.

## Data Availability

The datasets used and/or analyzed during the current study are available from the corresponding author on reasonable request.
